# Salt tolerance response revealed by RNA-Seq in a diploid halophytic wild relative of sweet potato

**DOI:** 10.1038/s41598-017-09241-x

**Published:** 2017-08-29

**Authors:** Yan Luo, Robert Reid, Daniella Freese, Changbao Li, Jonathan Watkins, Huazhong Shi, Hengyou Zhang, Ann Loraine, Bao-Hua Song

**Affiliations:** 1 Department of Biological Sciences, University of North Carolina at Charlotte, Charlotte, NC 28223 USA; 2 Key Laboratory of Tropical Forest Ecology, Xishuangbanna Tropical Botanical Garden, Chinese Academy of Sciences, Mengla, Yunnan 666303 China; 3 Department of Bioinformatics and Genomics, University of North Carolina at Charlotte, Charlotte, NC 28223 USA; 4 Breeding group, Monsanto Company, St. Louis, MO 63141 USA; 5 Department of Geography & Earth Sciences, University of North Carolina at Charlotte, Charlotte, NC 28223 USA; 6 Department of Chemistry and Biochemistry, Texas Tech University, Lubbock, Texas 79409 USA

## Abstract

Crop wild relatives harbor exotic and novel genetic resources, which hold great potential for crop improvement. *Ipomoea imperati* is a wild diploid relative of sweet potato with the capability of high salinity tolerance. We compared the transcriptomes of *I*. *imperati* under salt stress vs. control to identify candidate genes and pathways involved in salt response. *De novo* assembly produced 67,911 transcripts with a high depth of coverage. A total of 39,902 putative genes were assigned annotations, and 936 and 220 genes involved in salt response in roots and leaves, respectively. Functional analysis indicated a whole system response during salt stress in *I*. *imperati*, which included four metabolic processes: sensory initiation, transcriptional reprogramming, cellular protein component change, and cellular homeostasis regulation. We identified a number of candidate genes involved in the ABA signaling pathway, as well as transcription factors, transporters, antioxidant enzymes, and enzymes associated with metabolism of synthesis and catalysis. Furthermore, two membrane transporter genes, including vacuole cation/proton exchanger and inositol transporter, were considered to play important roles in salt tolerance. This study provided valuable information not only for understanding the genetic basis of ecological adaptation but also for future application in sweet potato and other crop improvements.

## Introduction

Soil salinity is one of the most severe constraints on global crop production. With the combined pressures of climate change and human population growth, salt tolerance is becoming an important agronomic trait to investigate for sustaining or even increasing the world’s food supply in marginal and high saline soils^1^. Extensive genetic and molecular studies on salt tolerance in glycophytic model plants and major crops such as *Arabidopsis thaliana*, rice, wheat, cotton and tomato^2, 3^, as well as in a number of halophytic species^4–6^, have been promising for elucidating molecular mechanisms of salt adaption. Hundreds of potential salt-responsive genes have been identified and analyzed to determine their expression patterns under salt treatments. These studies reveal molecular mechanisms in response to salt stress, including the salt overly sensitive (SOS) pathway, hormone signaling pathways, ion homeostasis, Reactive oxygen species (ROS) homeostasis, and osmotic regulation^7–9^.

Crop wild relatives typically have more genetic variations than domesticated crops, and thus provide plant breeders with a broader pool of potentially useful genetic resources^10, 11^. With the recent enormous progress in next generation sequencing technology, crop wild relatives have become a useful resource for developing salt and drought cultivars in many crops^12–14^. By the use of wide-hybridization and/or transgene techniques, several salt tolerance traits have been transferred from wild relatives into cultivated wheat and barley, and salt tolerance new cultivars have been successfully produced^15, 16^. Recently, wild halophyte rice, *Porteresia coarctata*, also has been used to improve rice salt tolerance^17, 18^.

Despite the important role of sweet potato (*Ipomoea batatas*) in global food and energy security, its traits improvement has been limited, including salt tolerance. Attempts to improve sweet potato salt tolerance have been made by introducing salt-tolerant candidate genes, such as, *iron-sulfur cluster biosynthesis* (*NFU1*) and *pyrroline-5-carboxylate reductaserelates* to proline metabolism (*P5CR*)^19, 20^. However, salt tolerance is a complex trait that has evolved independently by different mechanisms in diverse lineages^21^. Therefore, those salt tolerant candidate genes obtained from genetically distant species might not be effective in sweet potato. More study is needed to identify new markers and genes that can confer salt tolerance in sweet potato.

Sweet potato itself is salt sensitive, while its close wild relative, *I*. *imperati*, is a halophytic plant species^22^. *I*. *imperati*, known as beach morning glory, is a pantropical sandy coastal species. It grows in the sandy dune of coastal beaches and can toleratesaline and nutritionally poor soil conditions^23^. To our knowledge, few studies have investigated this species, and information about salt tolerance physiology, morphology, and molecular mechanism in beach morning glory is lacking. Interestingly, unlike most other halophytes, *I*. *imperati* lacks a salt gland or other specialized morphological traits to exclude salt. The adaptation to saline environments may be more exclusively attributed to the alteration in gene regulation^2, 4, 24^, which makes *I*. *imperati* extremely attractive for investigating its gene regulation strategies under salt stress.

In this study, we aimed to identify the candidate genes and pathways involved in response to salinity stress, using RNA-Seq analysis from the roots and leaves of *I*. *imperati*. The phenotypic and physiological responses to salt stress were studied under salt treatments and control at different time points. The transcriptomes from plants watered with saline or fresh water were compared to identify salt-responsive genes. Ultimately, the influence of crosstalk between gene expression and physiological responses were investigated to understand beach morning glory tolerance to salt stress and adaptation to saline habitats. The identified salt-responsive genes in the beach morning glory will shed light on the genetic basis of its ecological adaptation and thus they can be applied to developing salt-tolerant sweet potato cultivars.

## Results

### Phenotypic and physiological responses to salt stress in I. *imperati*

#### Plant growth

**Figure 1:**
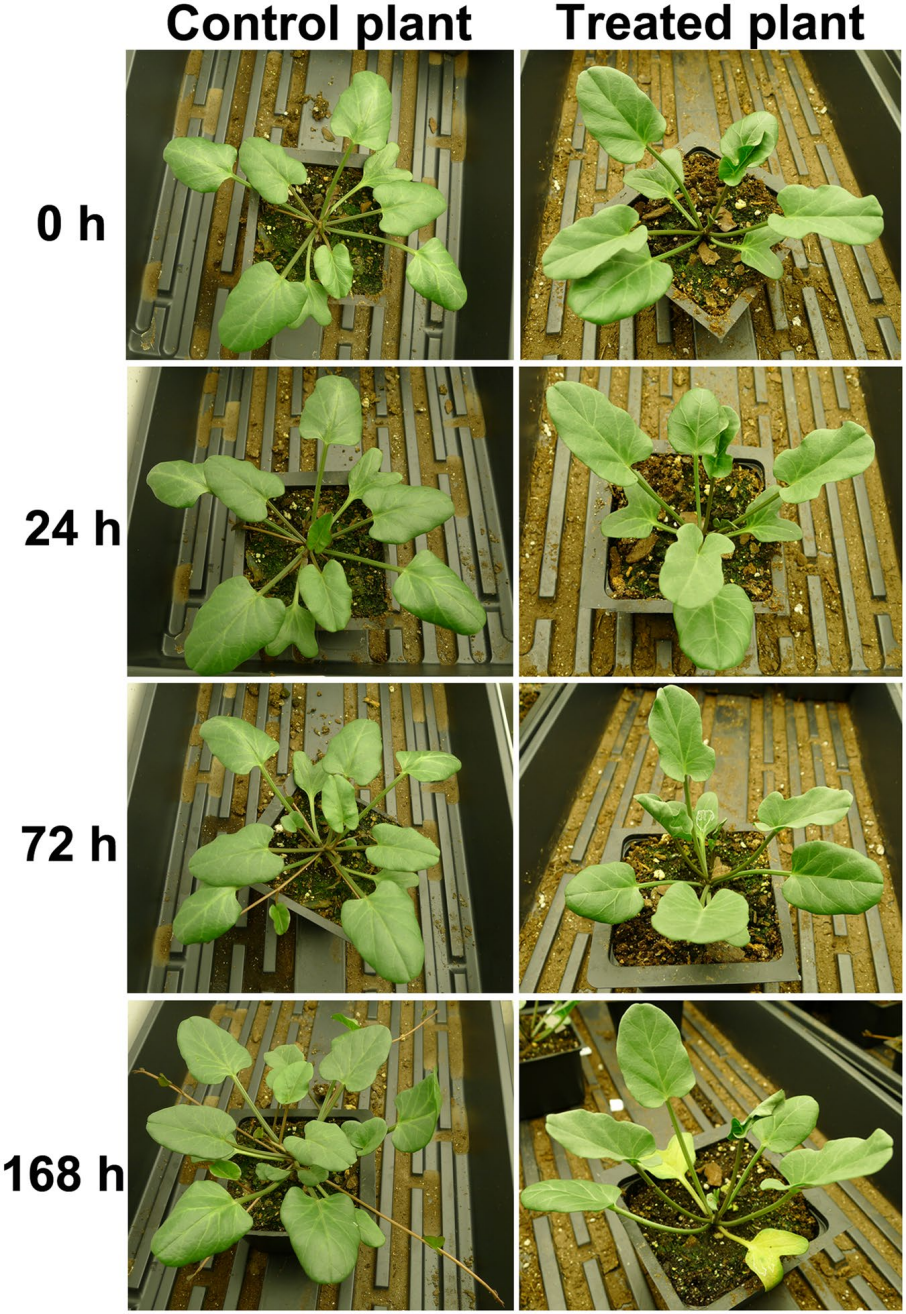
Comparison of control (fresh water) and salt-treated (600 mM NaCl) plants of *I*. *imperati* at 0 h, 24 h, 72 h (3d) and 168 h (7d).

After the 5-days pre-treatment with serials of NaCl from 100 mM to 500 mM, the pre-treated plants were daily applied with 600 mM NaCl. There was little difference in appearance between the control and the pre-treated plants (shown as 0 h in Fig. 1), with the control plants growing healthily with 7 leaves, and the pre-treated plants growing a little slowly with 6 leaves. After one day treatment with 600 mM NaCl, we observed a slightly delayed development in the treated plants compared with control plants (Fig. 1); The new 8^th^ leaf was developed in the control plants, while no new leaf emerged in the treated plants. After salt treatment for 72 h, in contrast to the control plants showing more new leaves and stolons, the treated plants developed relatively fewer new leaves and some were slightly curled without wilting. After salt treatment for 168 h (7 d), the control plants showed healthy growth with additional new leaves and stolons developed, while the treated-plants showed delayed development and the first-developed leaves turned yellowish and curled, but no wilting leaves were observed (Fig. 1). In summary, the treated plants showed relatively delayed growth in the treated plants than those control ones; however, no serious damage was observed under the high salinity condition (600 mM), such as leaf wilt or dehydration, suggesting a high salt tolerance capacity of beach morning glory.

#### Ion content

**Figure 2:**
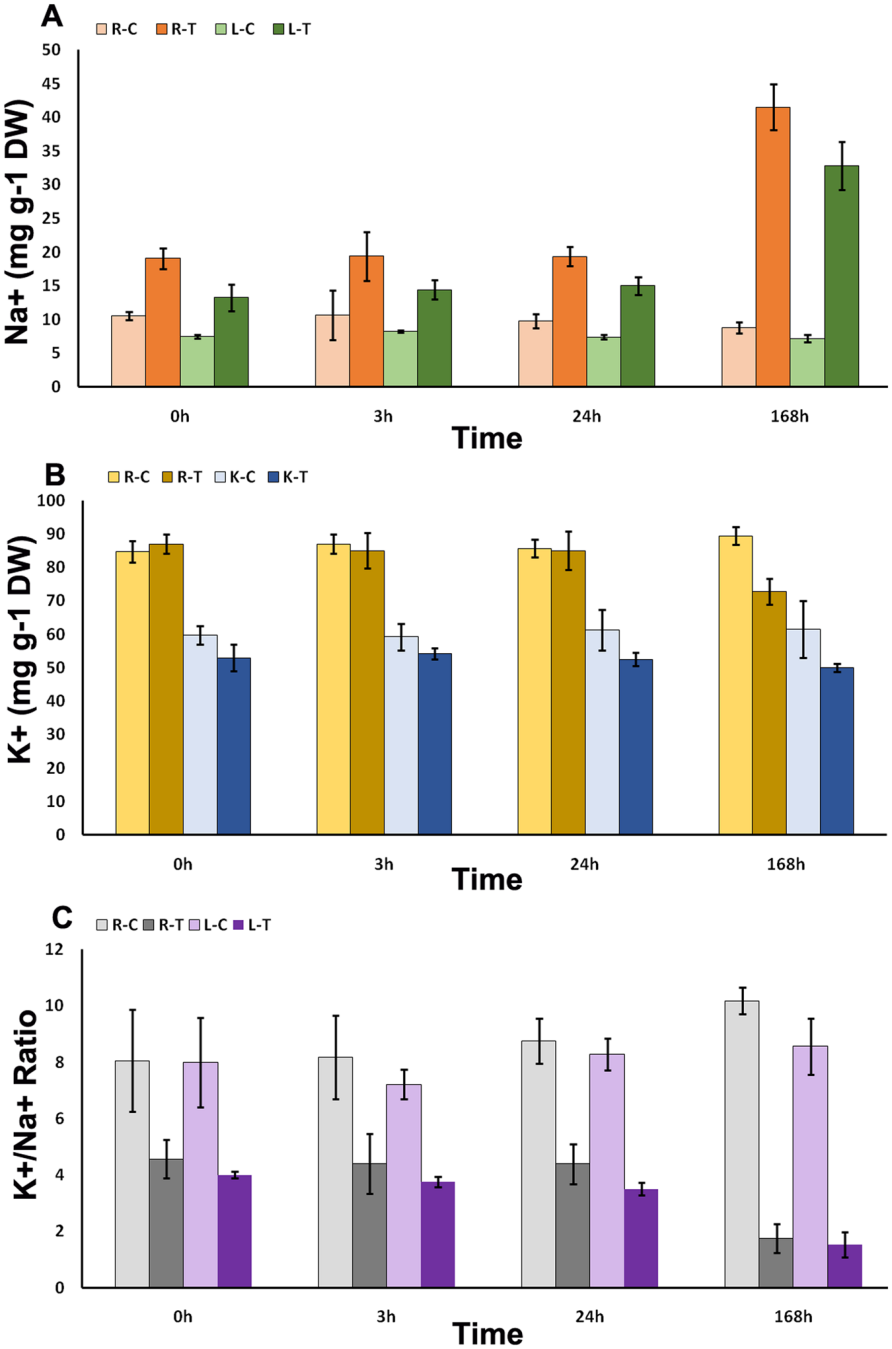
Ion content in control and salt-treated root and leave tissues of *I*. *imperati*. (**A**) Na^+^ content in control and salt-treatment roots and leaves. (**B**) K^+^ content in control and salt-treatment roots and leaves. (**C**) K^+^/Na^+^ Ratio in control and salt-treatment roots and leaves. R-C = control roots; R-T = salt-treatment roots; L-C = control leaves; L-T = salt-treatment leaves. Error bars represent SD.

Na^+^ and K^+^ contents were measured in both roots and leaves (Fig. 2). The Na^+^ significantly increased in both leaves and roots during the salt stress, with equal 2-fold increases observed in the first day including 0 h, 3 h, and 24 h, and higher 4-fold increases observed at 168 h. There was no significant change in K^+^ content within one-day treatment in root, but there was a slight decrease after 168 h. The K^+^ content decreased in leaves, in equal amounts at four time points. The content of K^+^ did not change much during salt treatment, suggesting the uptake and transport of K^+^ was not significantly affected in *I*. *imperati* under high salinity conditions. There was little difference in K^+^/Na^+^ ratio between roots and leaves, with a significant decrease compared with the controls, and a dramatic decrease in 168 h. The K^+^/Na^+^ ratio did not significantly vary in 0 h, 3 h, and 24 h, suggesting that there might exist a rapidly responsive ion regulation mechanism to maintain K^+^ and Na^+^ homeostasis during early salt stress.

### Processing and mapping of Illumina reads

A total of 12 cDNA libraries were sequenced. RNA-seq analyses were performed on three independent biological replicates (each replicate represents pooled tissues collected from different time points) of each sample in this study (L-C, Leaf control; L-T, Leaf treatment; R-C, Root control; R-T, Root treatment). The reads generated by the Illumina Hiseq. 2500 were initially processed to remove adapter sequences and low-quality reads. Supplemental Table S1 summarizes the assembly metrics for each of the conditions. 252,166,154 reads from all root and leaf tissues were filtered removing ~20% of total sequence, and roughly 20 Gb of nucleotides were obtained. All sequences regardless of condition were pooled for assembly in an effort to make a suitable background reference. *De novo* transcript assembly produced a total of 94,728 transcripts. Redundant sequences were removed by clustering sequences via CD-HIT, resulting in 67,911 remaining sequences (Table S1).

### Gene annotation

Conceptual translation of sequences produced 50,688 putative protein sequences, with 58.76% (39,902) annotated by a BLAST search against the NCBI non-redundant (NR) protein database. There are 1463 sweet potato proteins in the NCBI non-redundant (NR) protein database. We found 1297 genes (88.6%) with homologous matches (identity >60%) to sweet potato. The most commonly occurring matches were to genes from *Solanum lycopersium*.

### Functional classification

Following their NR annotations, we mapped protein sequences onto the records from the GO database and retrieved 21,418 GO annotations. Blast2Go assigned 18,034 with terms of “Biological process”, 13,322 with terms of “cellular component”, and 17,134 with terms of “molecular functions” (Fig. S1). Among the “Biological process” category, the two most abundantly represented terms were “metabolic process” (13,120, 61.2%), and “cellular process” (11,815, 55.2%). There were also a large number of genes involved in “single-organism process” (9,608, 44.9%), “biological regulation” (4,040, 18.9%), and “response to stimulus” (3,140, 14.7%) (Figure S2). In the “Cellular component” category, most protein sequences were located in “cell” (9,099, 42.5%), “cell part” (9,095, 42.5%), “organelle” (6,826, 31.9%) and “membrane” (5,134, 24.0%). In the “Molecular function” category, “catalytic activity” (11,386, 53.2%) and “binding” (10,787, 50.4%) were predominantly represented. “Catalytic activity” was mainly represented by genes for “transferase activity”, “hydrolase activity”, and “kinase activity”, while “binding” was mainly represented by genes with “nucleotide binding” and “protein binding”.

### Identification of differentially expressed genes in leaves and roots under salt stress

**Figure 3:**
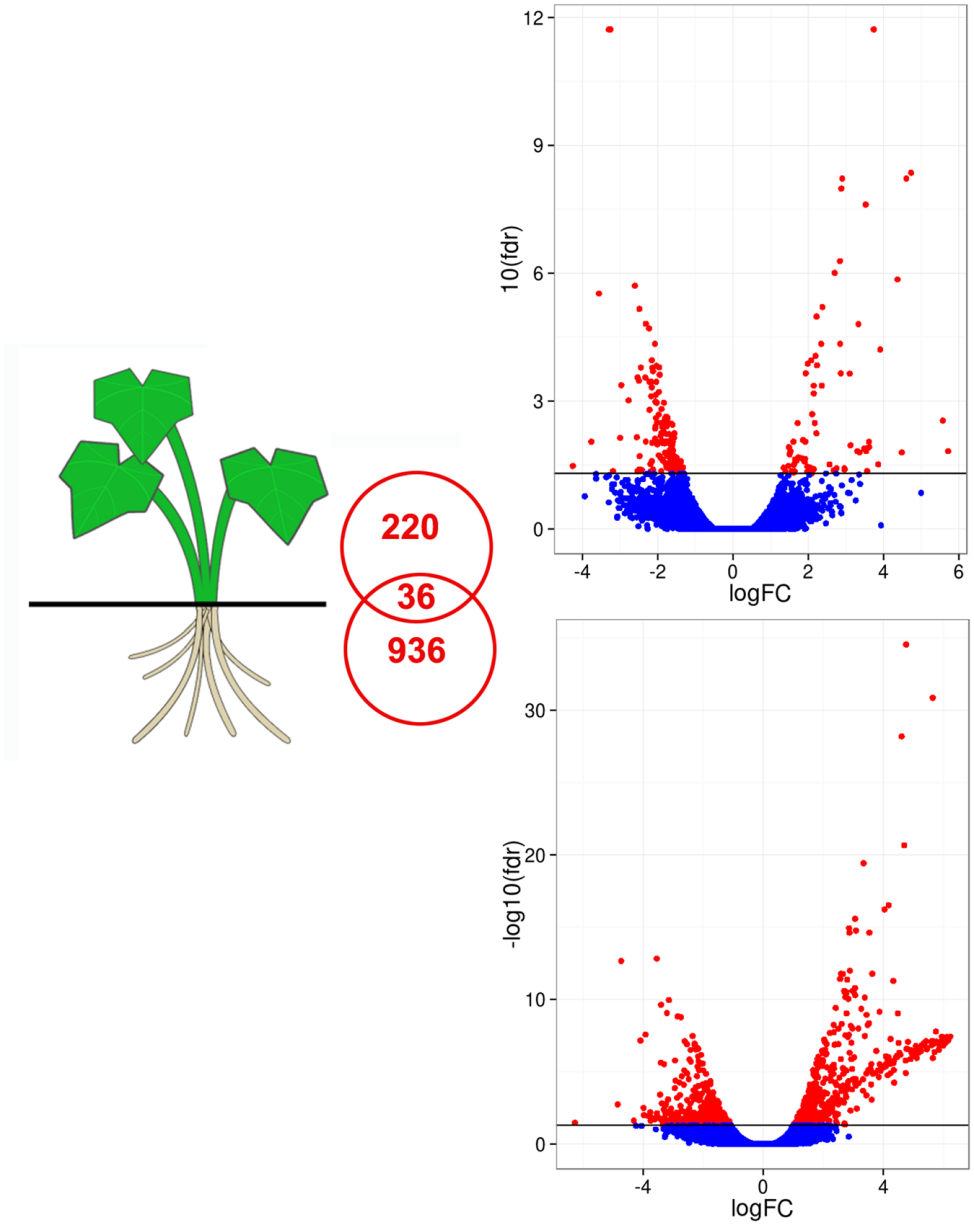
Volcano plots of transcriptomes for salt-treated root and leaf tissues in halophyte *I*. *imperati*. For each plot, the x-axis represents log base 2 fold-change, and the y-axis represents −log base 10 FDR. (**a**) *I*. *imperati* leaf; (**b**) *I*. *imperati* root. The horizontal bars on all figures represents FDR = 0.05, red dots equate to gene expression with FDR <0.05, and blue dots are gene expression values with FDR > 0.05.

A cutoff FDR value ≤ 0.05 and |Log_2_ fold change (FC)| ≥ 1 was used for identifying differentially expressed genes (DEGs). 936 DEGs were found in roots and 220 DEGs in leaves under salt treatment (Fig. 3). Among those, only 36 DEGs were shared in the two tissue types, while 96.2% and 83.6% tissue-specific DEGs in roots and leaves, respectively. Interestingly, there were more DEGs in roots than in leaves, implying that roots might undergo more complicated transcriptional regulation under salt treatment. In roots, the number of upregulated DEGs was greater than downregulated DEGs (544 upregulated and 392 downregulated). In leaves, there were more downregulated DEGs than upregulated DEGs (75 upregulated and 145 downregulated).

A total of 500 (53.4%) and 129 (58.6%) DEGs in roots and leaves, respectively, were annotated by Blast2Go. Although the number of DEGs differs between roots and leaves, the GO categories classifications were similar (Fig. S2). For example, some of the DEGs in roots and leaves shared GO terms, such as “Biological process”, “cellular process”, “metabolic process”, “single-organism process”, “biological regulation”, and “response to stimulus”. There were only two exceptions between the tissue types. The leave DEGs included some genes annotated with the term “signaling” but none of the DEGs from the roots had this term, while the root SEDs included genes annotated with the term “cellular component organization or biogenesis” but none of the DEGs from the leaf has this term. The significantly represented GO terms of “Cellular component” in DEGs of both roots and leaves were “intracellular”, “intracellular part”, “intracellular organelle”, “membrane-bounded organelle”, and “intrinsic component of membrane”. The only one exception was that “plasma membrane” represented in roots but not in leaves. The “Molecular function” categories were the same between roots and leaves, including “ion binding”, “organic cyclic compound binding”, “heterocyclic compound binding”, “oxidoreductase activity”, and “transferase activity binding”.

### GO enrichment analysis in DEGs

**Figure 4:**
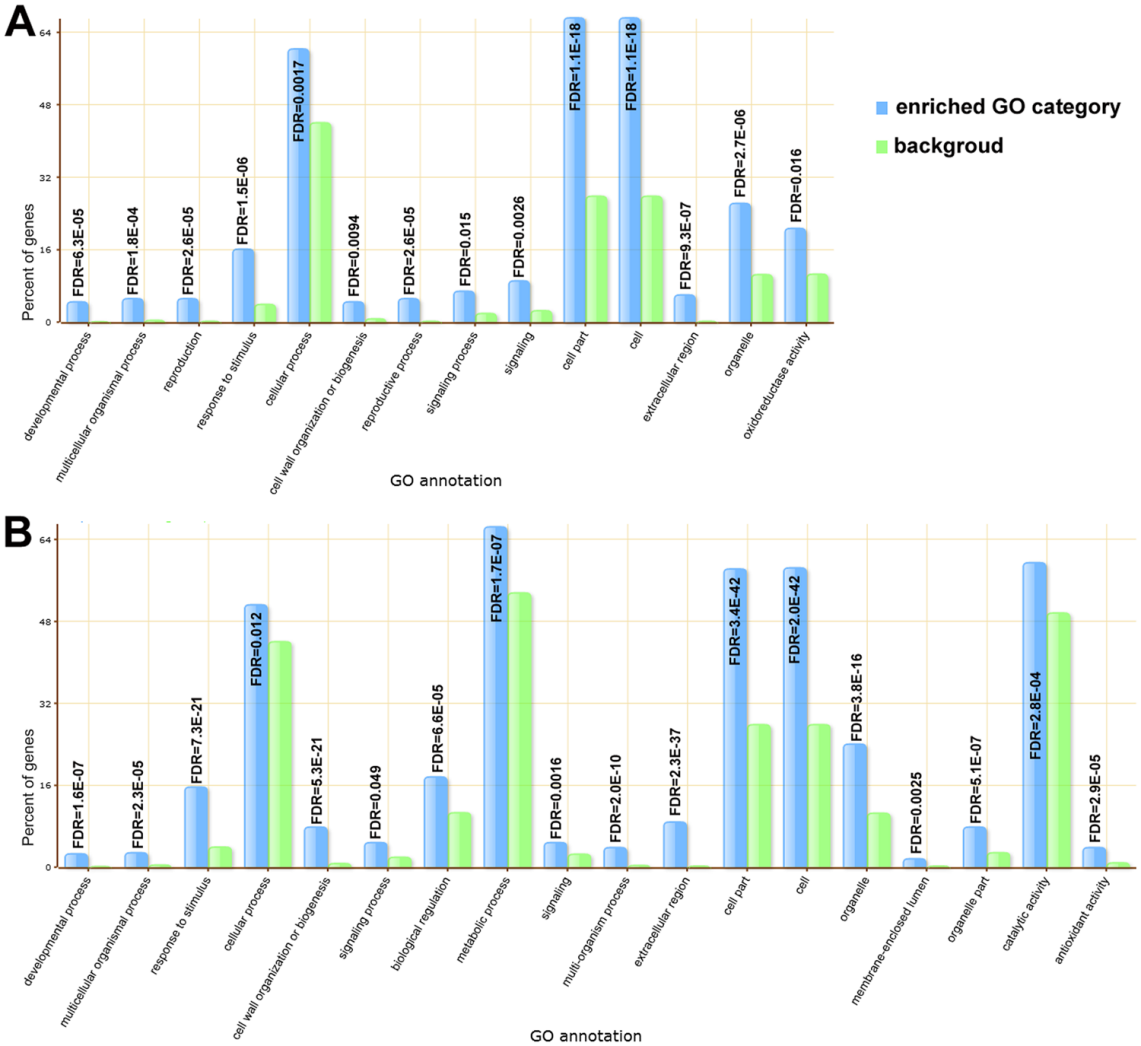
Enriched distributions of DEGs into GO categories (y-axis) according to GO enrichment analysis. x-axis: GO annotations; y-axis: percent of genes. Significance was determined by false discovery rate (FDR). (**A**) Leaf; (**B**) Root.

To further understand the expression changes in these two tissues, we conducted GO enrichment analysis for the DEGs with the tomato genome database as the background using AgriGO. In roots, there were 18 enriched GO terms, including 10 of biological process, 6 of cellular components and 2 of molecular functions (Fig. 4). In leaves, there were 14 enriched GO terms, including 9 of biological process, 4 of cellular components and one of molecular functions (Fig. 4). Responses to stimulus, cell, and cell part were significantly overrepresented both in leaves and roots, while there were more categories overrepresented in root. Metabolic processes, biological regulation, catalytic activity and antioxidant activity were all activated in roots but not in leaves. Oxidoreductase activity may be activated in leaves more than roots. “Response to stimulus” (GO:0050896) was a significantly enriched GO term in both leaves and roots (Fig. S3). GO enrichment analysis reveals an enrichment of genes involved in plant responses to abiotic stimuli, external stimuli, chemical stimuli, organic substances, defense response, as well as to hormone stimuli both in leaves and roots. More significant GO terms related to “response to stimulus” were enriched in roots (Fig. S3), such as response to oxidative stress, response to inorganic substance, response to biotic stimulus, all of which imply that a more complicated physiological response occurs in roots than that in leaves during salt stress. Both roots and leaves showed a similar gene expression pattern in response to salt stress, but different gene families within the same categories were activated. For example, GO category “response to hormone stimulus” (GO:0009725) was enriched in both leaves and roots, however, only five DEGs were assigned to five GO categories in leaves, including GO:0009725, GO:0009734, GO:0009737, GO:0009738 and GO:0010104, while 22 DEGs were assigned to twelve GO categories in roots, including seven hormone stimulus GO categories (Table S2).

To provide further insight on which plant pathways play a role during salt stress response, we performed a pathway level analysis using GAGE. We first align the 39,902 gene sequences to the potato RefSeq proteins to identify genes with known KEGG identifiers. The reason to choose potato is that it is the most closely related species available in RefSeq. BLASTX alignment produced 22,672 unique matches (identifiers). GAGE uses Log_2_ transformed FPKM values as expression levels to determine which pathways were active. This type of analysis differs from a gene level analysis such as GO, as a whole pathway was considered. Table 1 shows the most significantly induced pathways in roots.

**Table 1:** Top KEGG pathways identified by GAGE in root under salt stress. 91 different pathways were detectable, of which the top 10 are depicted here.

KEGG ID	Pathway
04712	Circadian rhythm - plant
01110	Biosynthesis of secondary metabolites - Reference pathway
03030	DNA replication
00650	Butanoate metabolism
03440	Homologous recombination
00073	Cutin, suberine and wax biosynthesis
03420	Nucleotide excision repair
00900	Terpenoid backbone biosynthesis
03040	Splicosome
00920	Sulfur metabolism

### Candidate DEGs involved in response to salt stress

Based on function annotation, DEGs were allocated into several biological processes, involved in ion homeostasis, ROS scavenging, hormone metabolism, signal transduction, amino metabolism, carbohydrate metabolism, and transcription factor regulation. Table S3 highlights the most significant DEGs based on functional categories that were candidate salt responsive genes.

DEGs include components of known salt-related signaling pathways, such as *Protein Phosphatase 2C* (*PP2Cs*), *serine/threonine kinases* (*SnRKs*), which were critical components of the ABA-signaling pathway, and *EIN2* and *EIN3* which participate in the ethylene signaling pathway. Some genes encoding for Receptor-like Kinase (RLK) were found, such as *LRR receptor-like serine/threonine-protein kinase*, receptor-like protein kinase *HAIKU2-like*.In our study, 36 Transcription Factors (TFs) were identified. The most abundant classes included *AP2/EREBP*, *bHLH*, *HD-ZIP* and *MYB*. The existence of various TFs in roots responding to salt stress was noteworthy.

We found many transcripts that encode a diversity of ion transporters including cation/H+ antiporter, vacuolar cation/proton exchanger, and root-specific metal transporter. We also found DEGs associated with solute transporters that were either upor downregulated during salt stress. These transporters included sugar transporters, amino acid transporters, and peptide transporters.

A number of antioxidant enzymes were detected involved in ROS scavenging during salt stress. Most of these antioxidant enzymes genes were highly activated in roots but not in leaves after salt treatment. 11 members of *POX* family were upregulated, 9 members of the *glutathione S-transferase* (*GST*) genes were significantly upregulated, and three *respiratory burst oxidase* homologs (*RBOHs*) genes were significantly downregulated. In addition, we found highly differentially expressed genes in roots that were involved in cell wall organization or biogenesis including *xyloglucan endotransglucosylase/hydrolase*, *cellulose synthase*, *expansin* genes and *pectin methylesterase*. Genes from the biosynthesis of cutin and wax pathway were also identified, as well as various stress-responsive proteins, such as osmotin-like protein and *Heat Shock Proteins*. Moreover, some genes involving in plant-pathogen interaction were also identified significantly upor down regulated, such as *chitinase*, *pathogenesis-related protein*.

### Validation of the RNA-Seq and gene expression analysis by real-time qRT-PCR

**Figure 5:**
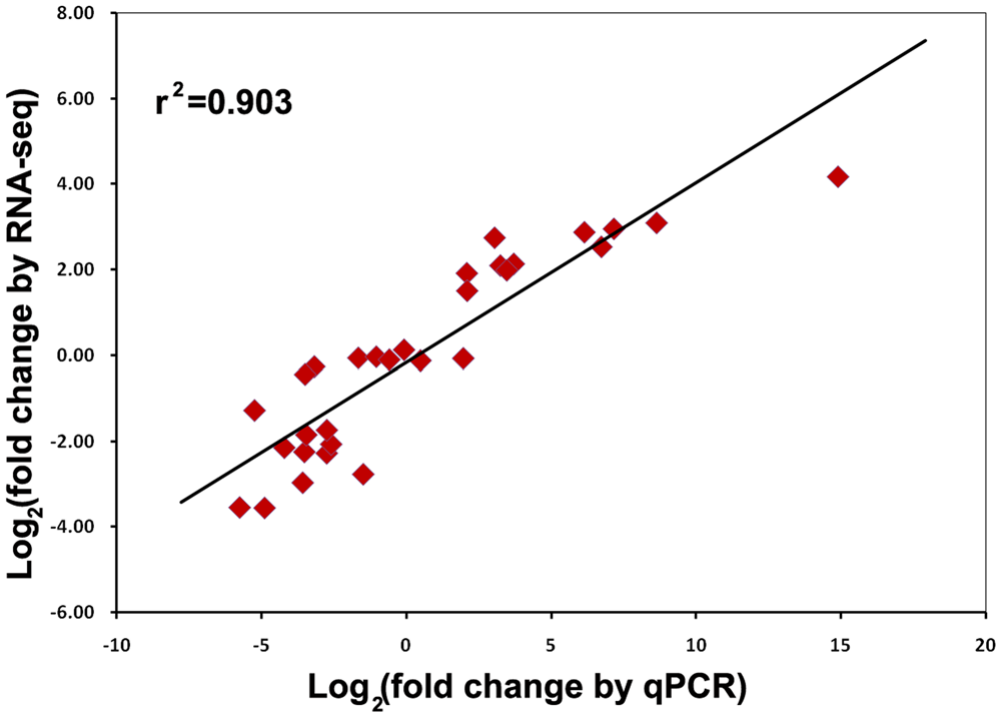
Validation of differential gene expression results obtained by RNA-Seq. The correlation of gene expression results obtained from qRT-PCR analysis and RNA-Seq for 15 random selected genes is shown.

Fifteen DEGs were randomly selected from a range of expression values for qRT-PCR analysis using the biological replicates of the RNAs previously used for RNA-Seq (Table S4). The analysis revealed a similar expression pattern of all the selected genes in real-time PCR analysis as observed from RNA-Seq data (R^2^ = 0.903) (Fig. 5). The validation experiments support the reliability of the relative values provided by the RNA-Seq analysis.

**Figure 6:**
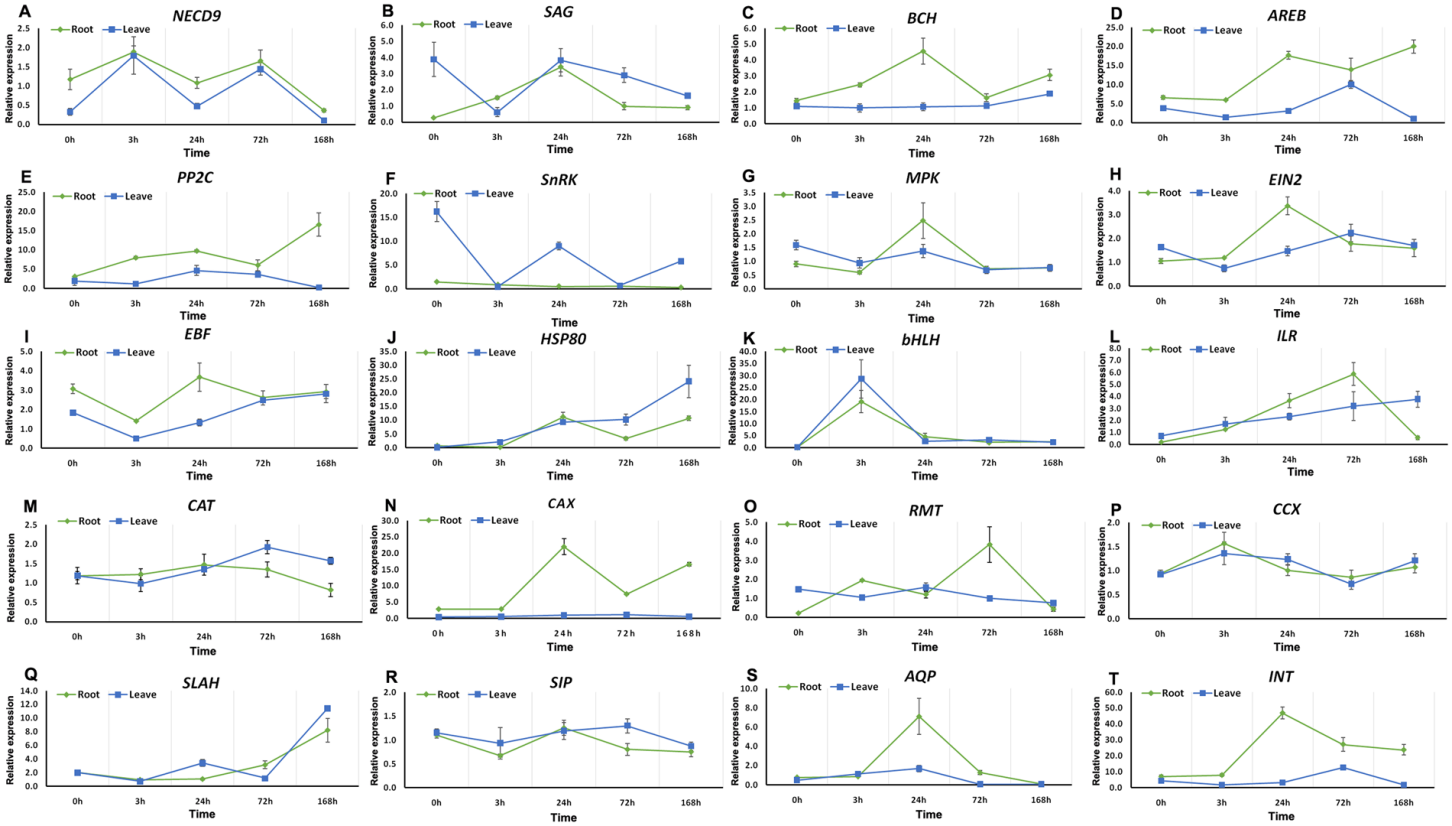
Selected expression profiles were validated with quantitative qRT-PCR in roots (green line) and leaves (blue line) of *I*. *imperati*. x-axis, five yantime point. y-axis, relative expression represented by mean fold changes of expression obtained from three biological replicates. Error bars represent SD.

Furthermore, twenty candidate genes involved in salt tolerance were further investigated by qRT-PCR at different time points to confirm the expression profiles under salt stress in both leaves and roots (Table S4). In most cases, the expression changes were more dramatic in roots than in leaves (Fig. 6). For roots, the selected DEGs were significantly upregulated at 3 h and/or 24 h post-salt stress implying that the expression pattern in roots changed rapidly after salinity stress. Among eight transporter transcripts, the transcripts from *CAX* and *INT* genes (Fig. 6N,T), show a dramatic change in roots. High salinity results in a dramatic increase in gene expression at 24 h, with the expression of *CAX* and *INT* increasing by 22-fold and 53-fold respectively. The transcripts associated with ABA signaling pathway and ABA biosynthesis were found upregulated in roots and/or in leaves (Fig. 6A–F). Transcription factors *bHLH* and *ILR3* all show upregulation in both leaves and roots, with the highest expression of *bHLH* at 3 h and the highest expression of *ILR3* at 72 h (Fig. 6K,L).

## Discussion

**Figure 7:**
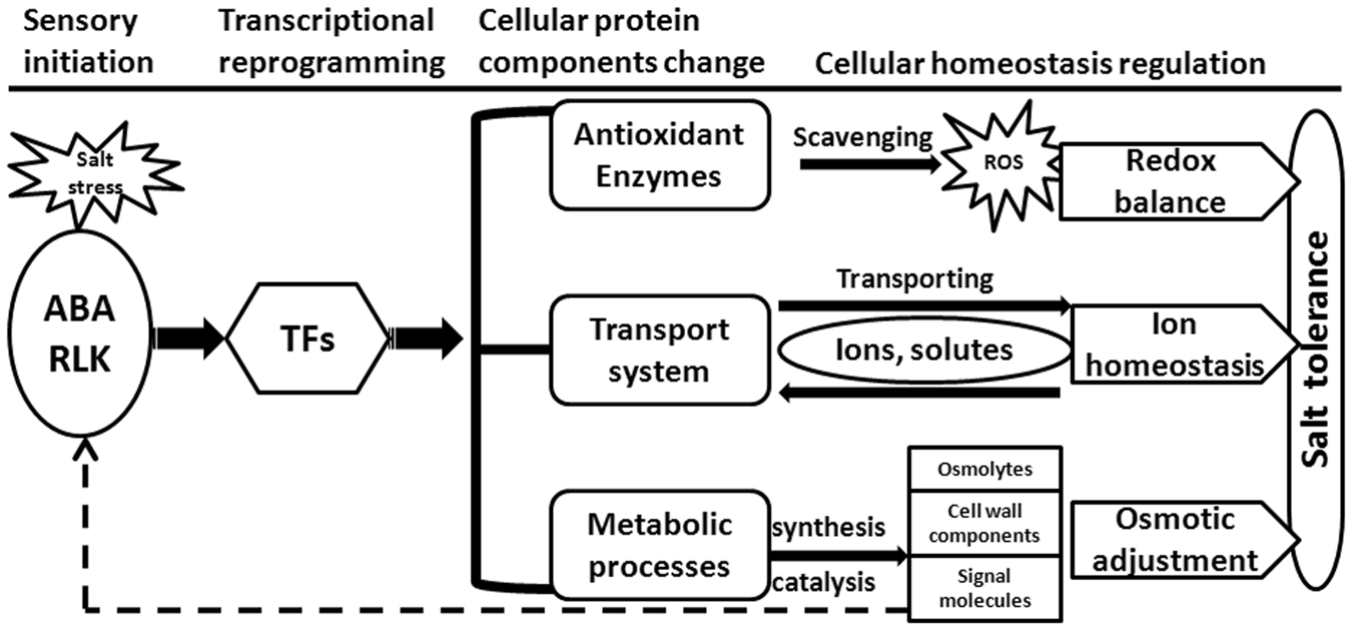
Schematic summaries of the whole response system activated in response to salt stress in *I*. *imperati*. Salt stress is first perceived by ABA signaling pathway or RLKs, then the activated signaling pathway regulates transcriptional level reprogramming, resulting in gene expression and cellular protein components alteration. The activation of cellular homeostasis mechanisms for ion homeostasis, osmotic balance and redox balance: ABA, ABA signaling pathway; RLK, receptor-like kinase; TFs, transcription factors; ROS, reactive oxygen species.

The overall salt tolerance of a plant is determined by integrated effects of many different mechanisms^9, 25^. Under high salt treatment (600 mM NaCl), *I*. *imperati* mobilizes many genes related to a variety of biological processes as part of a whole cellular responsive system. As summarized in Fig. 7, the whole responsive system is composed of four separate and dependent processes, including sensory system initiation, transcriptional reprogramming, cellular protein components change, and cellular homeostasis regulation.

Signals of salt stress can be detected by receptors and transmitted by signaling pathways that trigger transcriptional reprogramming by stress-activated transcription factors^26, 27^. Sensory system and signaling pathways play a key role in salt tolerance^7, 28^. Studies focusing on glycophytes have revealed the involvement of several signal transduction pathways in salt stress response, including SOS pathway^29, 30^, the ABA signaling pathway, the ethylene signaling pathway, the calcium signaling pathway, as well as the *receptor-like kinase* (*RLK*)^31–34^. Our study suggests that hormones might play an important role in salt signaling in *I*. *imperati*, including differentially expressed hormone receptors such as auxin receptor, ethylene receptor and ABA receptors (Table S3). The involvement of an ABA pathway also seems plausible. All four components were differentially expressed in roots, including ABA sensor *PYL*, negative regulator *PP2C*, positive regulators *SnRK* and *AREB*. Also, a number of signaling proteins, such as *LRR receptor-like serine/threonine-protein kinase*, were upregulated under salt stress.

Accordingly, we saw a change in cellular protein components including various enzymes involved in synthesis and catalysis processes, various transporters, and antioxidant enzymes (Fig. 7). Many metabolic processes were activated in *I*. *imperati*, including catabolic process, carbohydrate metabolic process, small molecule metabolic process, and oxidation reduction. Activations of these metabolic processes or pathways might be involved in the production of salt-induced ROS or signal molecules, and synthesis of stress-related metabolites, such as hormone, multiple osmolytes, cell wall components, etc. ref. 35. The transport system was also highly activated with transcripts encoding a diversity of transporters showing differential expression. Previous studies show antioxidative response mechanisms were effective in ROS homeostasis, especially in halophytes^5, 6^. The antioxidant defense system was markedly influenced in *I*. *imperati* with many genes associated with antioxidant enzyme activity showing different expression patterns under salt stress.

Many of these active proteins mobilize metabolic processes and structural changes that participate in the maintenance of osmotic, ion and redox balance (Fig. 7). For example, ion transporters including cation/H^+^ antiporter, vacuolar cation/proton exchanger, root-specific metal transporter, etc., may be responsible for selective accumulation of ions, control of ion uptake and compartmentalization^36, 37^. Cellular osmotic balance related salt tolerance relies on many kinds of compatible solutes and small molecule osmoprotectants, such as proline, inositol, sugars and betaines^38^. The solute transporters including sugar transporters, amino acid transporters, and peptide transporters, may be responsible for regulating cellular osmotic balance. The *POX* and *GST* gene families in roots might function in protecting *I*. *imperati* against oxidative damage.

Further investigation of the strategies used for salt tolerance in beach morning glory reveals a few halophytic-specific genes that might contribute to salt tolerance. Two genes encoding cation transporter (*vacuolar cation/proton exchanger*, *CAX*), and sugar transporter (*inositol transporter*, *INT*) were significantly upregulated (2 to 50-fold) during salt stress. These two genes thus might be important in the response to high salinity in *I*. *imperati* (Fig. 6N and T).

Cation transporters play major roles in maintaining ion homeostasis^9, 39^. The Na^+^ transporters NHX-type transporter were involved in Na^+^ accumulation in the vacuoles during salt exposure and have been identified in many plants^40–42^. It is interesting that *NHX* genes, well-studied in *Arabidopsis* and other species, were detected but did not show significantly differential expression between salt-treated plants vs. control plants in our study. This indicates that *NHX* genes might express constitutively high without a need of further activation during salt stress in this species. This can be tested by the comparison of their expression differences between beach morning glory vs. a glycophyte relative without salt stress. Further comparative gene expression investigation between *I*. *imperati* and its glycophytic relatives is needed. We found an alternative cation transporter gene encoding a putative vacuolar cation/proton exchanger, showing significant upregulation in salt-treated roots. This gene belongs to another cation transporter, *cation/proton exchanger* (*CAX*) family. *CAX* antiporter located in both the plasma membrane and vacuolar membrane is thought to be an important member for ion homeostasis in plant cells, but only a small number of *CAX* genes have been functionally characterized^43^. *CAX* antiporters transport multiple cations out of plasma membrane into the vacuole. *CAX* was involved in ion regulation and has been identified from a few plants^44, 45^. Luo *et al*.^46^ found that a soybean *GmCAX1* could improve salt tolerance in transgenic *Arabidopsis*. We found that *CAX* gene expressions were maintained at high levels during salt stress, reaching a dramatic 20-fold increase (Fig. 6N). This cation transporter gene might contribute to the process of Na^+^ and K^+^ homeostasis.

The accumulation of inositols and/or its methylated derivatives were found in several halophytic species under salt stress, suggesting an important role for these compounds in salt tolerance^47, 48^. The importance of INT in stress signaling is known in the common ice plant (*Mesembryanthemum crystallinum*). In this well-studied halophyte, Myo-inositol (MI) accumulation and enhanced *INT* show as an adaptive osmoregulatory response to salt. Also, sodium uptake and accumulation to a high concentration in vacuoles were dependent on the presence of MI^49, 50^. Chauhan *et al*.^51^ found that MI transport in the tonoplast of *M*. *crystallinum*actually acts as a Na^+^/myo-inositol symporter system, removing Na^+^ from root vacuoles. We discover a putative *INT* gene significantly upregulated both in roots and leaves, and higher expression in roots than that in leaves. The expression of *INT* was 52-fold upregulated within 24 h (Fig. 6T), suggesting a rapid response to high salinity stimuli. Moreover, activation of several genes in the inositol synthesis pathway occurs during salt stress in some halophytic species^49, 52^. In the common ice plant, *myo-inositol 1-phosphate synthase* (*INPS1*) was upregulated in leaves^49, 52^. A few studies have demonstrated that engineering of some genes, such as those involved in inositol metabolic pathway in the halophytic wild rice *Porteresia*, can confer salinity tolerance in cultivated rice^53^. In contrast, *Arabidopsis* showed no upregulation of these genes or increases in inositol-derived compounds, which suggested that the strategy of osmoprotection was some what different between glycophyte and halophyte^52^. In our study, *inositol-3-phosphate synthase* (comp40237_c0_seq. 1, *INPS3*) was shown highly upregulated in leaves but not in roots based on the transcriptomes of this species, suggesting that the inositol synthesis might be activated in leaves in response to salt stress. Further studies are needed to investigate the functions of inositol transporter and inositol biosynthesis genes in *I*. *imperati*.

In recent years, various halophytic species have been used as sources to improve salt tolerance in many crops^3, 12^. However, conventional breeding aimed at trait improvement has been limited in sweet potato due to its large genome size, hexaploidy, male sterility, and self-incompatibility. There is potential to deliver substantial improvement in salt tolerance from its close wild halophytic relatives. In this study, we utilized a closely-related diploid wild-relative of sweet potato, *I*. *imperati*, which offers distinct salt tolerance capacity as a genetic resource to study salt adaptation and improve salt tolerance in sweet potato. Using transcriptome analysis by RNA-Seq, we successfully identified 67,911 putative genes in this species. Among these sequences, 1120 genes were differentially induced by salt stress. These salt-responsive genes with various functions such as ion transport, osmolytes, signal transduction, antioxidant enzymes, biosynthesis enzymes (Table S3), as we discussed above, might play key roles in conferring salt tolerance in *I*. *imperati*. Most of those genes involved in salt tolerance mechanisms may be evolutionarily conserved and important for conferring salt tolerance in different plant lineages^54^. The strategies used for salt tolerance in beach morning glory are partly in accordance with the hypothesis that salt tolerance mechanisms are largely conserved in halophytes and glycophytes, and that the large variations in their tolerance arise from subtle differences in the regulation of the same basic set of genes^2^. On the other hand, this *de novo* RNA sequencing assembly approach allowed us to explore novel roles of known genes as well as assess the roles of new genes, so-called orphan genes, taxonomically distinct genes that lack a distinguishable homology to unrelated species, in salt tolerance^55^. Orphan genes often contribute novel, lineage specific functionality under extreme abiotic and biotic stresses^56^. In the present study, there were 28,009 (41.24%) putative genes identified in *I*. *imperati* that have no matches to the NCBI sequence database. Of these unknown sequences, 129 were upregulated and 148 were downregulated, respectively. Although it is difficult to discern if these sequences encode functional proteins without further investigation, the high levels of expression following salt stress suggest that these orphan genes may play important roles in conferring salt tolerance^57^. This highlights the potential to discover novel species-specific genes and pathways in beach morning glory.

In conclusion, we characterized a comprehensive transcriptome of a wild halophytic sweet potato, *I*. *imperati*, using the Illumina sequencing technology. The results suggest that extensive genetic regulation under salt stress is responsible for salt tolerance in this halophytic species. The candidate salt-responsive genes identified in *I*. *imperati* contain both previously reported salt-responsive genes and novel genes that can be used as a new resource for genetic engineering in sweet potato or other crops. Meanwhile, a great number of orphan genes might have the great potential to discover novel species-specific or lineage specific salt tolerance mechanisms. The transcriptome analyses presented in this study not only provide plenty of bioinformatics data but also expand the vision of the genetic basis underlying adaptation for beach sandy dune.

## Materials and Methods

### Plant material and salt stress conditions

Seeds of *I*. *imperati* were collected from St. George Island, Florida, USA. All seeds were scarified before germination. The germinated seeds were transferred to soil in pots, and grown at 28 °C under long day conditions in a 16 h light/8 h dark cycle in an environmental chamber (Model E-41L2, Percival Scientific, USA) with 50% humidity and 600 micromoles/m^2^/s light intensity. For salinity stress treatment, two–weeks-old seedlings were watered with NaCl solution from 100 mM to 500 mM for 5 days to acclimate to salt soil. On day 6, 600 mM NaCl was applied daily for 7 days. Meanwhile, seedlings watered with freshwater were used as controls. Three biological replicates of roots and leaves from both treated (600 mM NaCl) and control (fresh water) plants were harvested at 0 h, 3 h, 24 h, 3 days, and 7 days after the treatment, respectively. All samples were flash frozen in liquid nitrogen and stored at −80 °C for RNA extraction.

### Ion content measurement

Control and salt-treated leaves and roots were harvested at 0 h, 3 h, 24 h and 7 days. Tissues were rinsed with deionized water and dried at 65 °C for 2 days.50 milligrams of dried leavesandrootsmaterial was milled to powder, then extracted with 5 ml of 0.1 M HNO_3_ for 90 min at 95 °C and then filtered through Whatman filter paper. Na^+^ and K^+^ contents in the solutions were determined with a Varian Spectra AA-10 atomic absorption spectrophotometer.

### RNA extraction, cDNA library preparation, and RNA-Seq

Tissues from all time points (0 h, 3 h, 24 h, 3 days, and 7 days) were powdered and equal amounts of tissue (by weight) were pooled as one biological replicate for RNA preparation; three replicates were prepared for treatment and control resulting in 12 RNA libraries. The pooled samples were denoted as L-C (Leaves control), L-T (Leaves treated), R-C (Roots control) and R-T (Roots treated) according to the leaves and roots with salt or water treatments. Total RNA was extracted using RNeasy Mini Kit (Qiagen, USA) following the supplied protocol and treated with DNase I (Thermo Scientific, USA). The quantity and integrity of the total RNA were determined using an Agilent 2100 bioanalyzer (Agilent, USA), and found to be RIN >9. The 12 RNA libraries were prepared using Illumina TruSeqRNA sample Preparation Kit (Illumina, USA). The quality of cDNA libraries were determined by applying the DNA 1000 chip on the Bioanalyzer 2100 (Agilent, USA). Twelve normalized cDNA libraries were constructed and sequenced using the Illumina Hiseq. 2500 platform (North Carolina State University) to generate 100 bp paired-end reads.

### Transcriptome *de novo* assembly, annotation and differentially gene expression analysis

Sequence reads were filtered using the Fastx-toolkit (http://hannonlab.cshl.edu/fastx_toolkit/) for quality and adapter sequences were removed using the fastq_quality_trimmer. Trimmed reads were combined across all conditions and *de novo* assembled via Trinity^58^ using default settings in order to build a suitable set of reference contigs. Trinity contigs of high similarity were clustered into groups with CD-HIT-EST and a single representative from each cluster was used as a reference sequence for read alignment. For annotation purposes, sequences from each CD-HIT cluster were transdecoded into predicted proteins using Transdecoder (http://transdecoder.github.io)^59^. Only proteins greater than 100 amino acids in length were retained for further annotating. Sequences were initially annotated by blasting nucleotide sequences against the NCBI NR database using BLASTX. Sequences were annotated with Gene Ontology categories (GO, http://www.geneontology.org) using Blast2Go^60^. Read count tables were produced via SAMtools^61^, from each biological replicate, the transcript abundance of each gene was estimated by fragments per kilobase of exon per million fragments mapped (FPKM). FPKM values were calculated and RSEM was used for counting the mapped reads. Expression levels of each tissue were compared between the treated and control samples to find DEGs using the edgeR R package^62^. A false-discovery rate (FDR) of 0.05 and |Log_2_ fold change| ≥ 1were used as the threshold to identify differentially expressed genes (DEGs).

### Differentially expressed genes abundance analysis

The DEGs were subjected to GO enrichment analysis using AgriGO software (http://bioinfo.cau.edu.cn/agriGO/)^63^ (cutoffs: FDR <0.05) with Singular Enrichment Analysis (SEA) performing. DEGs from the roots or leaves were the input list, while the reference background was tomato genome database (*Solanum lycopersicum* iTAG2.3), which is a closely related species.

To identify relevant KEGG pathways, we aligned the sequences to an annotated NCBI RefSeq dataset of a closely related species, potato (*Solanum tuberosum*, downloaded from NCBI’s FTP site ftp://ftp.ncbi.nih.gov/genomes/refseq/plant/Solanum_tuberosum/representative/GCF_000226 075.1_SolTub_3.0/). Sequences were aligned via Blastx to assign a Refseq ID annotation. These IDs along with their corresponding read count data (FPKM values) were subjected to GAGE analysis after log transformation^64^ to identify potential differentially expressed pathways. GAGE performs gene set enrichment by assigning KEGG IDs to each matching Ref-Seq hit and then identifies and ranks the most enriched pathways based on fold change differences between conditions. Identified pathways were then visualized using Pathview^65^.

### Validation of RNA-seq and gene expression analysis by real-time PCR (qRT-PCR)

In order to prove the reliability of the RNA-Seq data, qRT-PCR was performed on the same RNA pools that had been previously used for RNA-Seq. Fifteen DEGs, which were shown significantly differential expression in roots and/or leaves, were randomly selected for qRT-PCR analysis (Table S4). Furthermore, to verify the differential expression, qRT-PCR was performed on a new set of 3 replicates for each sample at different time points. A set of 20 DEGs in response to salt stress was chosen from different categories, including eight transcripts encoding transporters, six transcripts belonged to components of ABA signaling pathway and ABA biosynthesis, three transcripts belong to components of ethylene signaling pathway, two transcripts encoding transcription factors, and one transcript encoding a responsive protein (Table S4). The gene-specific primers were designed based on the sequencing data using the Primer-BLAST tool (http://www.ncbi.nlm.nih.gov/tools/primer-blast). The actin gene of *I*. *imperati* was used as an internal control. The gene description and abbreviation and primer sequences used in this study were listed in Table S4.

The first-strand cDNAs were synthesized from 1 ug of DNase-treated total RNA using RevertAid RT Reverse Transcription Kit (Thermo Scientific, USA). qRT-PCR was performed using ABI 7500 fast Real-Time PCR system (Applied Biosystems, USA) and SYBR Green FastMix (Quanta Biosciences, USA). Reactions were performed in triplicate and contained 200 ng of cDNA, 10 uM of each primer. Amplifications were performed under the following conditions: 95 °C for 5 min, followed by 40 cycles of 95 °C for 3 s, 60 °C for 30 s. Relative expression for each sample was calculated using the 2^−ΔΔCt^ methods with normalization to the internal control genes.

## Electronic supplementary material

Supplementary Figures

Supplementary Dataset 1

Supplementary Dataset 2

Supplementary Dataset 3
