# Supplementary material for: Salt tolerance response revealed by RNA-Seq in a diploid halophytic wild relative of sweet potato
Source: Sci Rep. 2017 Aug 29;7:9624. doi: 10.1038/s41598-017-09241-x (PMC5575116; doi:10.1038/s41598-017-09241-x)
Supplement: Supplementary file 1 — Supplementary Figures [file 41598_2017_9241_MOESM1_ESM.pdf]

Salt tolerance response revealed by RNA-Seq in a diploid halophytic wild relative of sweet potato

Yan Luo[1,2]*, Robert Reid[3]*, Daniella Freese[1, 3], Changbao Li[4], Jonathan Watkins[5], Huazhong Shi[6], Hengyou Zhang[1], Ann Loraine[3&], and Bao-Hua Song[1&]

1. Department of Biological Sciences, University of North Carolina at Charlotte, Charlotte, NC 28223, USA
2. Key Laboratory of Tropical Forest Ecology, Xishuangbanna Tropical Botanical Garden, Chinese Academy of Sciences, Kunming, Yunnan, 666303, China
3. Department of Bioinformatics and Genomics, University of North Carolina at Charlotte, Charlotte, NC 28223, USA
4. Breeding group, Monsanto Company, St. Louis, MO, 63141, USA
5. Department of Geography & Earth Sciences, University of North Carolina at Charlotte, Charlotte, NC 28223, USA
6. Department of Chemistry and Biochemistry, Texas Tech University, Lubbock, Texas 79409, USA

* These authors contributed equally to this project
[&]Corresponding authors:

Bao-Hua Song

Department of Biological Sciences,

University of North Carolina at Charlotte,

Charlotte, NC 28223

bsong5@uncc.edu

001-704-687-8670

Ann Loraine

Department of Bioinformatics and Genomics,

University of North Carolina at Charlotte,

Charlotte, NC 28223

aloraine@uncc.edu

Supplementary Figures

Figure S1. GO categories.

Figure S2. GO category of DEGs at the second level (A. Biological process categories; B. Molecular function categories; C. Cellular components categories) in roots and leaves in *I. imperati*. Different color blocks represent all kinds of GO categories. The number in color block indicates the DEG numbers in each GO term.

Figure S3. AgriGO analysis of DEGs enrichments of GO category (GO:0050896) “response to stimulus” in leaves (A) and roots (B). Each box shows the GO term number and GO term, an FDR in parenthesis. The pair numerals in the left represent the number of genes in input list associated with that GO term and number of genes in the input list. The pair of numerals in the right represents the number of genes associated with a particular GO term in the total *I. imperati* annotated genes. Box colors indicate levels of statistical significance: yellow=0.05; orange=1e ×10-5; and red=1e ×10-9.

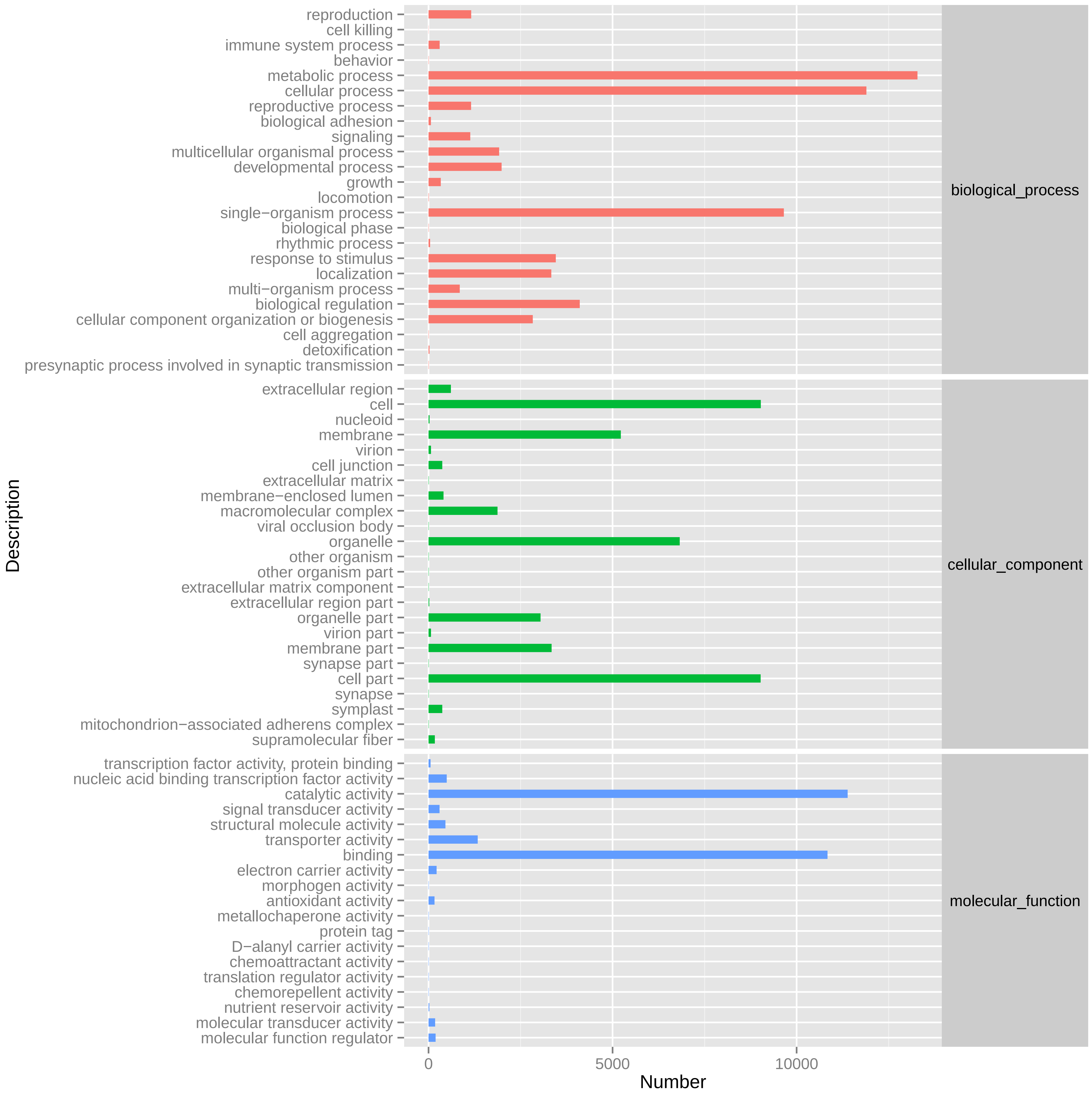

reproduction
cell killing
immune system process
behavior
metabolic process
cellular process
reproductive process
biological adhesion
signaling
multicellular organismal process
developmental process
growth
locomotion
single−organism process
biological phase
rhythmic process
response to stimulus
localization
multi−organism process
biological regulation
cellular component organization or biogenesis
cell aggregation
detoxification
presynaptic process involved in synaptic transmission
extracellular region
cell
nucleoid
membrane
virion
cell junction
extracellular matrix
membrane−enclosed lumen
macromolecular complex
viral occlusion body
organelle
other organism
other organism part
extracellular matrix component
extracellular region part
organelle part
virion part
membrane part
synapse part
cell part
synapse
symplast
mitochondrion−associated adherens complex
supramolecular fiber
transcription factor activity, protein binding
nucleic acid binding transcription factor activity
catalytic activity
signal transducer activity
structural molecule activity
transporter activity
binding
electron carrier activity
morphogen activity
antioxidant activity
metallochaperone activity
protein tag
D−alanyl carrier activity
chemoattractant activity
translation regulator activity
chemorepellent activity
nutrient reservoir activity
molecular transducer activity
molecular function regulator
biological_process
cellular_component
molecular_function
Description
0
5000
10000
Number

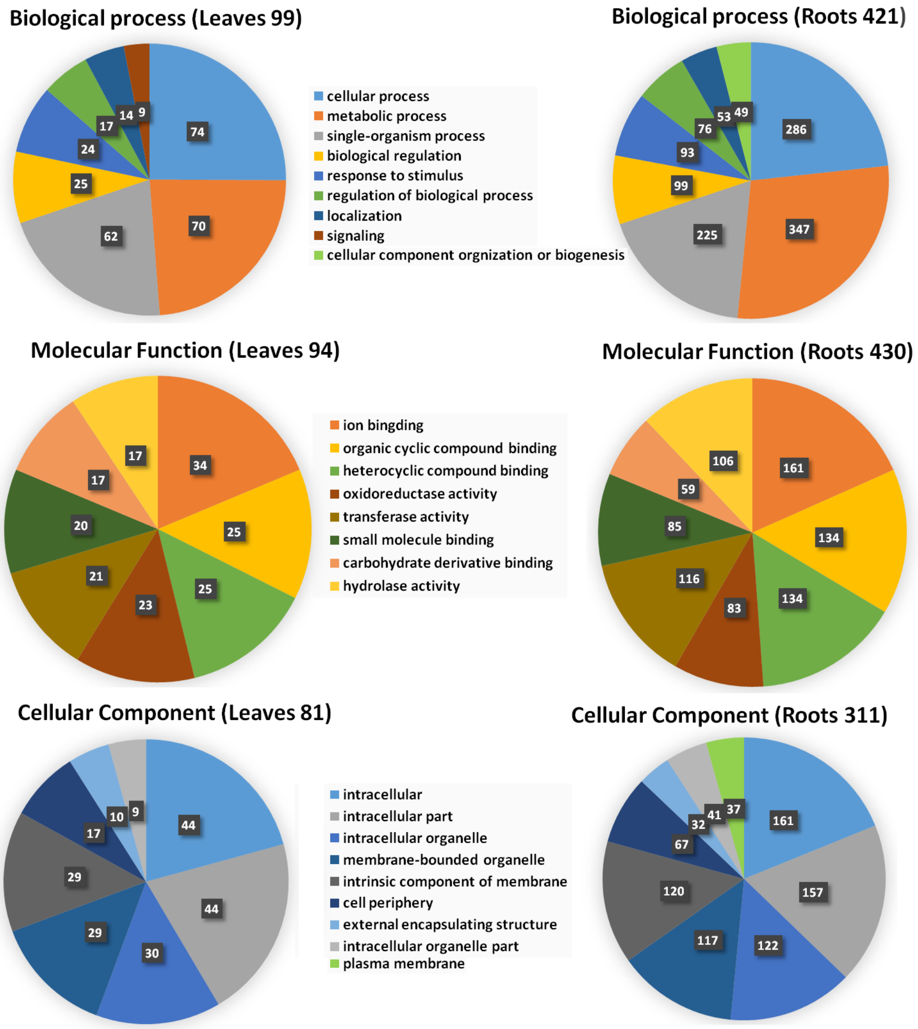

Biological process (Leaves 99)
74
70
62
25
24
17
14
9
Biological process (Roots 421)
286
347
225
99
93
76
53
49
cellular process
metabolic process
single-organism process
biological regulation
response to stimulus
regulation of biological process
localization
signaling
cellular component orgnization or biogenesis
Molecular Function (Leaves 94)
34
25
25
23
21
20
17
17
Molecular Function (Roots 430)
161
134
134
83
116
85
59
106
ion bingding
organic cyclic compound binding
heterocyclic compound binding
oxidoreductase activity
transferase activity
small molecule binding
carbohydrate derivative binding
hydrolase activity
Cellular Component (Leaves 81)
44
44
30
29
29
17
10
9
Cellular Component (Roots 311)
161
157
122
117
120
67
32
41
37
intracellular
intracellular part
intracellular organelle
membrane-bounded organelle
intrinsic component of membrane
cell periphery
external encapsulating structure
intracellular organelle part
plasma membrane

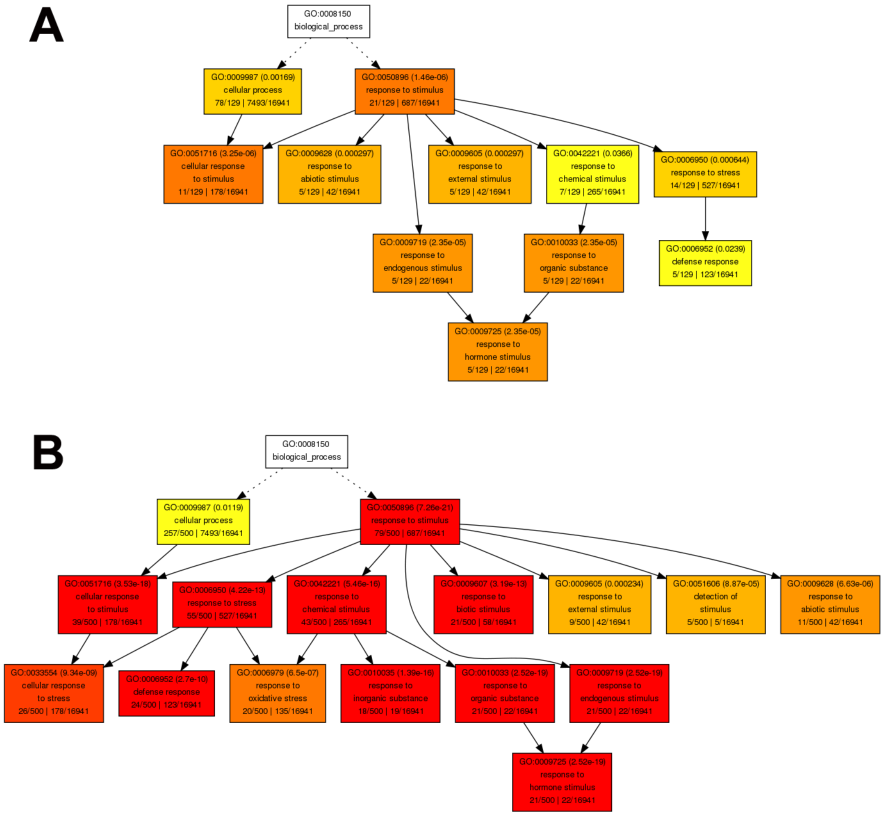
A
GO:0008150 biological_process
GO:0009987 (0.00169) cellular process 78/129 | 7493/16941
GO:0050896 (1.46e-06) response to stimulus 21/129 | 687/16941
GO:0051716 (3.25e-06) cellular response to stimulus 11/129 | 178/16941
GO:0009628 (0.000297) response to abiotic stimulus 5/129 | 42/16941
GO:0009605 (0.000297) response to external stimulus 5/129 | 42/16941
GO:0042221 (0.0366) response to chemical stimulus 7/129 | 265/16941
GO:0006950 (0.000644) response to stress 14/129 | 527/16941
GO:0009719 (2.35e-05) response to endogenous stimulus 5/129 | 22/16941
GO:0010033 (2.35e-05) response to organic substance 5/129 | 22/16941
GO:0006952 (0.0239) defense response 5/129 | 123/16941
GO:0009725 (2.35e-05) response to hormone stimulus 5/129 | 22/16941
B
GO:0008150 biological_process
GO:0009987 (0.0119) cellular process 257/500 | 7493/16941
GO:0050896 (7.26e-21) response to stimulus 79/500 | 687/16941
GO:0051716 (3.53e-18) cellular response to stimulus 39/500 | 178/16941
GO:0006950 (4.22e-13) response to stress 55/500 | 527/16941
GO:0042221 (5.46e-16) response to chemical stimulus 43/500 | 265/16941
GO:0009607 (3.19e-13) response to biotic stimulus 21/500 | 58/16941
GO:0009605 (0.000234) response to external stimulus 9/500 | 42/16941
GO:0051606 (8.87e-05) detection of stimulus 5/500 | 5/16941
GO:0009628 (6.63e-06) response to abiotic stimulus 11/500 | 42/16941
GO:0033554 (9.34e-09) cellular response to stress 26/500 | 178/16941
GO:0006952 (2.7e-10) defense response 24/500 | 123/16941
GO:0006979 (6.5e-07) response to oxidative stress 20/500 | 135/16941
GO:0010035 (1.39e-16) response to inorganic substance 18/500 | 19/16941
GO:0010033 (2.52e-19) response to organic substance 21/500 | 22/16941
GO:0009719 (2.52e-19) response to endogenous stimulus 21/500 | 22/16941
GO:0009725 (2.52e-19) response to hormone stimulus 21/500 | 22/16941
